# Physics-Informed Reconstruction of Transmural Myocardial Deformation from 3D Echocardiography: Validation Against Cardiac MRI

**DOI:** 10.1007/s10439-026-04335-y

**Published:** 2026-08-13

**Authors:** Satya Prakash Pradhan, Arash Yavari, Issac D. Lindley, Nader Binesh, Yuri P. Matusov, Kambiz Ghafourian, Gianni Pedrizzetti, Arash Kheradvar

**Affiliations:** 1 Department of Biomedical Engineering, Samueli School of Engineering, University of California, Irvine, CA 92697-2715, USA; 2 School of Civil and Environmental Engineering, Georgia Institute of Technology, Atlanta, GA 30332, USA; 3 The George W. Woodruff School of Mechanical Engineering, Georgia Institute of Technology, Atlanta, GA 30332, USA; 4 Department of Imaging, Cedars-Sinai Medical Center, 127 S San Vicente Blvd, Los Angeles, CA 90048, USA; 5 Division of Pulmonary & Critical Care Medicine, Department of Medicine, Cedars-Sinai Medical Center, 8670 Wilshire Blvd, Beverly Hills, CA 90211, USA; 6 Cedars-Sinai California Heart Center, 8670 Wilshire Blvd, Beverly Hills, CA 90211, USA; 7 Department of Engineering and Architecture, University of Trieste, Via Valerio 6/1, 34127 Trieste, Italy; 8 Department of Radiological Sciences, School of Medicine, University of California, Irvine, CA 92697-2715, USA

**Keywords:** Myocardial strain, Finite strain, Speckle tracking, Echocardiography, Cardiac MRI, Left ventricle

## Abstract

**Purpose:**

Quantitative assessment of myocardial deformation is increasingly important in clinical cardiology, yet conventional two-dimensional (2D) echocardiography and standard three-dimensional (3D) approaches remain limited by out-of-plane motion and incomplete characterization of transmural mechanics. To address these limitations, we introduce a physics-informed framework for 3D echocardiography that reconstructs the full finite strain tensor across the entire myocardial wall. As an initial methodological study, we demonstrate the framework and validate it against cardiac magnetic resonance in a small cohort.

**Methods:**

Endocardial and epicardial surfaces were segmented from 3D echocardiographic datasets and tracked throughout the cardiac cycle using speckle-tracking techniques. An optimization framework with a soft volumetric penalty was implemented, permitting volume change at finite cost while maintaining tracking fidelity and geometric smoothness. The resulting deformation field enabled reconstruction of the complete 3D strain tensor. Global strain measurements derived from the method were validated against cardiac magnetic resonance (CMR) measurements obtained in two subjects.

**Results:**

Global longitudinal and circumferential strain values obtained from the proposed framework showed strong agreement with CMR measurements. The optimization procedure also demonstrated robustness to segmentation variability and reduced errors associated with epicardial tracking. Beyond conventional strain indices, the method enabled reconstruction of spatially resolved principal strain fields throughout the ventricular wall, revealing physiologically consistent transmural gradients and contraction patterns.

**Conclusion:**

Physics-informed integration of speckle tracking with biomechanical constraints enables robust reconstruction of 3D myocardial deformation from echocardiography. This framework provides a comprehensive and physically consistent characterization of myocardial mechanics from widely available 3D echocardiographic data. These initial results support the feasibility of the proposed framework and motivate future evaluation in larger, more diverse patient cohorts to establish its clinical reliability.

## Introduction

The assessment of myocardial deformation has gained growing clinical importance in cardiology because it provides a direct measure of myocardial mechanical function. Myocardial strain imaging is highly sensitive for detecting subtle abnormalities in cardiac performance and can often identify dysfunction earlier than conventional metrics such as ejection fraction. Consequently, deformation indices—particularly global longitudinal strain—have been widely adopted in both clinical practice and research to evaluate myocardial performance in a wide spectrum of cardiovascular diseases [1, 2].

Traditionally, cardiac deformation has been assessed using two-dimensional (2D) imaging—most commonly echocardiography—where strain measurements are derived along directions visible within the imaging plane. As a result, these measurements are largely limited to myocardial shortening along the base–apex axis (longitudinal strain) or to deformation in the transverse plane (circumferential strain) [3]. Although these indices provide clinically valuable information, the complex ventricular motion resulting from the 3D architecture of myocardial fibers limits the completeness of deformation analysis derived from planar imaging approaches.

The myocardium is composed of a complex 3D arrangement of helical fiber bundles whose orientation varies transmurally across the ventricular wall. At the endocardial layer, myocardial fibers are organized in an oblique helical pattern aligned predominantly along the longitudinal axis of the ventricle. Moving toward the mid-myocardium, fiber orientation progressively transitions to a more circumferential configuration, while in the epicardial layer the fibers assume a helical orientation opposite to that of the endocardium [4, 5].

The complex architecture of myocardial fibers leads to a highly coordinated pattern of myocardial deformation during systole, characterized not only by longitudinal and circumferential shortening but also by ventricular torsion and transmural shear. These mechanical features arise from the 3D arrangement of myocardial fibers, whose orientations vary continuously across the ventricular wall from endocardium to epicardium. Although this architecture plays a fundamental role in ventricular mechanics, accurately mapping the true 3D fiber structure in vivo remains extremely challenging, if not currently infeasible, with standard clinical imaging techniques.

As a result, direct quantification of deformation along the true local fiber directions throughout the myocardium is difficult to achieve. A practical alternative is to estimate a surrogate direction of myocardial contraction that reflects the resultant of the collective contraction of fibers with varying orientations within the myocardial wall. This effective contraction direction captures the net mechanical action generated by the underlying fiber architecture and can therefore provide a meaningful representation of ventricular pumping function.

These complex mechanical interactions highlight the inherently 3D nature of myocardial deformation and cannot be fully captured using measurements confined to a single imaging plane. This limitation underscores the constraints of conventional planar strain analysis and emphasizes the potential advantages of 3D quantification of myocardial deformation [6, 7].

In recent years, advances in 3D echocardiography have enabled estimation of the full myocardial strain tensor, allowing simultaneous assessment of myocardial deformation along multiple directions. This capability provides a more comprehensive characterization of myocardial mechanical activity and offers new opportunities to better understand the complex mechanics of ventricular contraction under both physiological and pathological conditions [6, 8, 9].

Despite these advances, most currently available echocardiographic approaches are limited to evaluating deformation at the inner myocardial boundary (endocardium), which can typically be identified in 3D echocardiography and segmented with reasonable confidence. In contrast, the outer myocardial boundary (epicardium) is often less echogenic, making its delineation more challenging and prone to segmentation errors. Consequently, relatively few studies have attempted to quantify epicardial deformation [10], restricting the ability to characterize deformation across the full myocardial wall thickness. This limitation hinders a comprehensive assessment of myocardial mechanics and reduces the physiological interpretability of deformation measures derived from echocardiography.

To address this limitation, we have developed a method to estimate myocardial deformation using both endocardial and epicardial information derived from echocardiography. The proposed framework leverages the near-incompressibility of myocardial tissue as a soft biomechanical regularizer to improve epicardial delineation and to achieve a physically realistic representation of the myocardial wall throughout the cardiac cycle. By incorporating this regularization, the method enhances the robustness of epicardial tracking and enables deformation analysis across the entire myocardial thickness. The performance of the proposed framework is evaluated through comparison with 2D myocardial deformation measurements obtained from cardiac magnetic resonance (CMR), which is widely considered a reference modality for quantitative assessment of cardiac structure and function. The present work is intended as an initial methodological validation study: the framework is demonstrated and validated against CMR in a small cohort, with larger-cohort evaluation of clinical reliability left to future work.

The present work differs from existing 3D echocardiographic strain approaches in three key aspects:

- Reconstruction of a 3D deformation field across the full myocardial wall.
- Incorporation of a biomechanical regularizer based on the near-incompressibility of myocardial tissue.
- Estimation of the full finite strain tensor and principal strain directions.

## Methods

### Clinical Data Acquisition and Image Processing

#### Study subjects

This study was conducted under clinical protocols approved by the Institutional Review Boards of Cedars-Sinai Medical Center (STUDY00001683) and University of California, Irvine (Protocol #20216931). Although the present validation focuses on two subjects with concurrent CMR acquisition, the framework was developed and tested across multiple additional 3D echocardiography datasets to ensure robustness. Data from two participants were included: a 67-year-old Hispanic White male with normal left ventricular (LV) function and a 40-year-old Asian female diagnosed with Pulmonary Arterial Hypertension. For both participants, echocardiography and CMR examinations were performed on the same day. Heart rates recorded during CMR and echocardiography were closely matched: 63 bpm and 64 bpm, respectively, for the healthy subject, and 77 bpm and 82 bpm, respectively, for the patient with PAH. Written informed consent was obtained from all participants.

#### 3D Echocardiography Acquisition and Segmentation

Transthoracic 3D echocardiography was performed using a Philips EPIQ CVx system equipped with an X5-1 transducer (Philips Medical Systems, Andover, MA, USA). Full-volume 3D datasets of the LV were acquired over one cardiac cycle and stored in DICOM format for offline analysis.

**Fig. 1:**
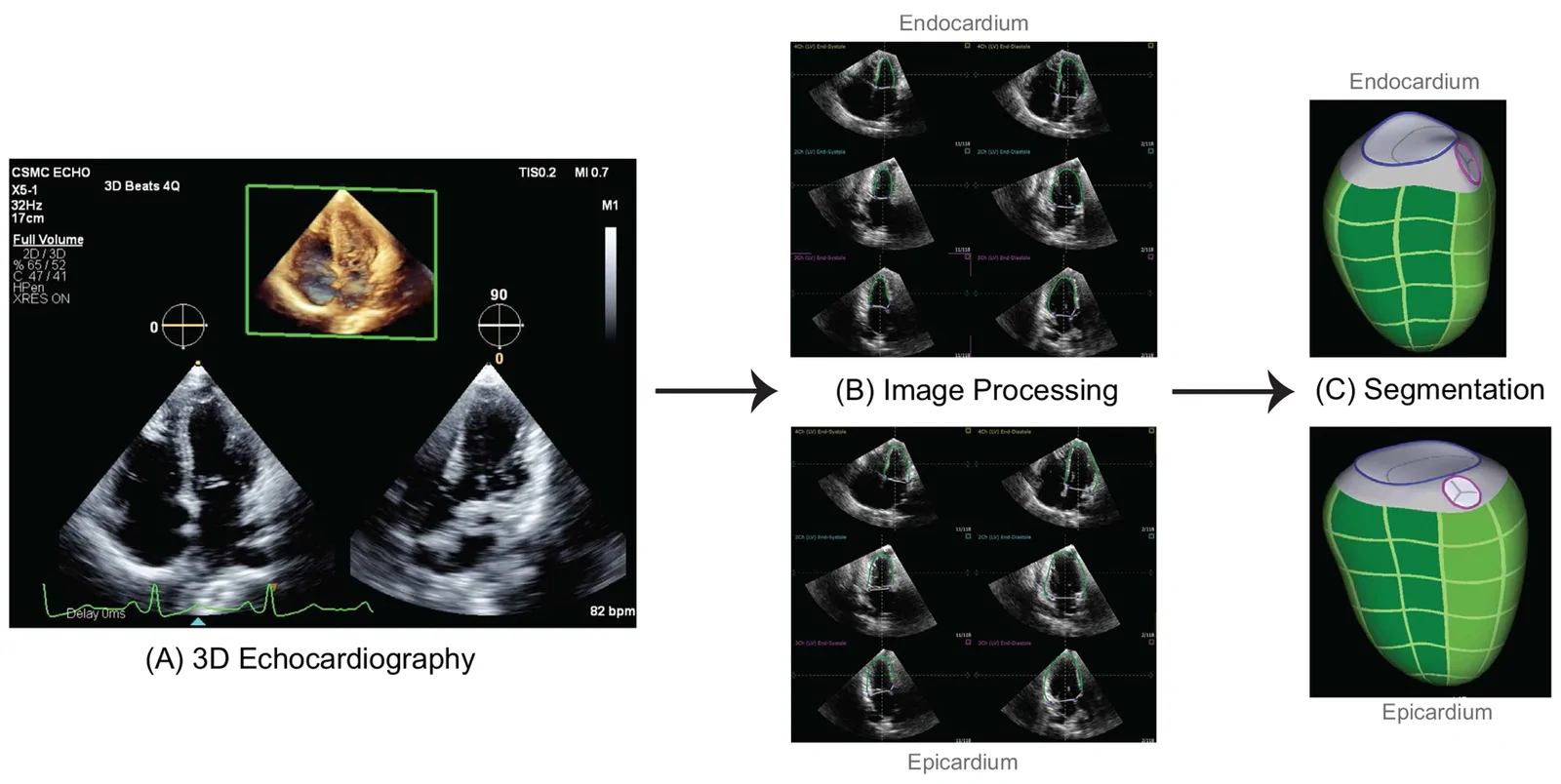
Workflow for LV segmentation: **A** 3D Echocardiography: Volumetric images captured using a Philips matrix-array probe by expert sonographers; **B** Clinical Image Processing: 3D segmentation and speckle tracking via TomTec-Arena (TTA); **C** Segmentation Results: Final volumetric endocardial and epicardial surfaces serving as the geometric basis for the proposed strain analysis

Post-processing of the DICOM datasets was performed using the *TomTec-Arena Ultrasound Workspace* (Philips Medical Systems, Andover, MA, USA). The *4D-LV-Analysis* module automatically generated a dynamic endocardial surface model and identified end-diastolic (ED) and end-systolic (ES) frames (Fig. 1). After manual refinement of the ED contours, the LV surfaces were propagated across the cardiac cycle using the built-in speckle-tracking algorithm. ES contours were subsequently reviewed and refined when necessary to ensure anatomical consistency, yielding physiologically coherent dynamic endocardial surface representations throughout the cardiac cycle.

Because the *TomTec-Arena Ultrasound Workspace* is primarily designed for endocardial segmentation, the epicardial surface was approximated by outwardly expanding the endocardial ED contours using the built-in LV mass estimation option. The resulting contours were then manually refined to match the visible myocardial thickness at ED. These epicardial ED contours were propagated throughout the cardiac cycle using the speckle-tracking algorithm, and the ES contours were subsequently adjusted manually based on careful visual assessment to ensure anatomical consistency.

Standard epicardial speckle tracking is inherently less accurate than endocardial segmentation due to the reduced contrast between the epicardial boundary and the surrounding tissues. To evaluate the robustness of the proposed optimization procedure under such uncertainty, intentional perturbations of the epicardial border were introduced. Specifically, the original end-systolic (ES) epicardial contour was systematically under-contoured (contracted) and over-contoured (expanded), generating three distinct borders for subsequent processing.

#### CMR Acquisition

To enable cross-modality comparison and validation, both subjects underwent standard clinical CMR imaging on the same day as their echocardiography examinations. CMR was performed on a 1.5T clinical MRI scanner (Magnetom Sola, Siemens Healthineers, Erlangen, Germany) using retrospectively ECG-gated, breath-held 2D balanced steady-state free precession cine sequences.

**Fig. 2:**
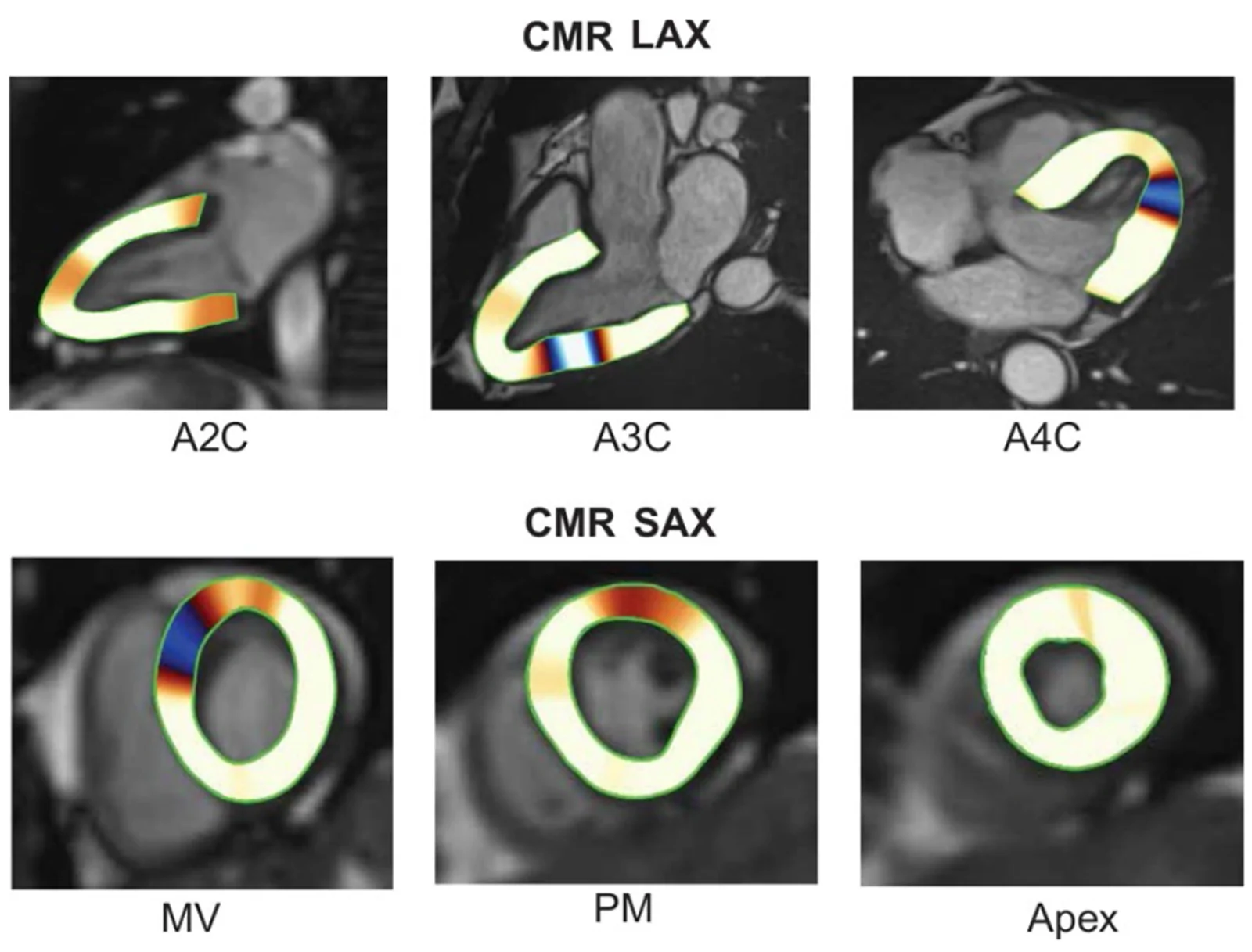
End-systolic CMR segmentations performed in *Medis Suite MR*, displaying long-axis (A2C, A3C, and A4C) and short-axis (mitral valve, papillary muscles, and apex) cross-sections

The CMR protocol included three long-axis (LAX) views (two, three, and four chambers) and three short-axis (SAX) slices acquired at the basal, mid-ventricular (papillary muscle), and apical levels (Fig. 2). LAX images were obtained with a slice thickness of 6.0 mm and an in-plane spatial resolution of 1.8 × 1.8 mm, whereas SAX images were acquired with an 8.0 mm slice thickness and an in-plane spatial resolution of 1.3 × 1.3 mm. To ensure sufficient temporal resolution for comparison with echocardiography-derived measurements, cine images were reconstructed into 30 cardiac phases per R–R interval.

### Optimization Method

Myocardial tissue deforms in a nearly, but not strictly, incompressible manner. Although its solid constituents and perfusing blood are individually close to incompressible, the expulsion of blood from the wall permits a measurable, regionally variable volume change over the cardiac cycle [11–16]. The local volume change is quantified by the Jacobian *J*, the ratio of deformed to reference volume, with *J* = 1 for locally isochoric deformation. Echocardiography-derived reconstructions depart from local volume preservation largely because the endocardial and epicardial surfaces are segmented and tracked independently, so that the apparent volume change is dominated by segmentation noise and tracking error rather than by genuine physiology, particularly at the less echogenic epicardium. We therefore treat near-incompressibility as a soft regularizer rather than a hard constraint, drawing the less reliable epicardial surface toward volume consistency with the more reliable endocardial segmentation while permitting physiological volume change.

The reconstruction is posed as the unconstrained minimization of a composite objective that balances three terms: a volumetric regularizer penalizing deviations from local volume preservation, a data-fidelity term that maintains consistency with the speckle-tracking motion, and a geometric smoothness term. The problem is formulated as the minimization of a composite objective function:

(1) $$E_{\text{total}}=\alpha_{\text{vol}}E_{\text{vol}}+\alpha_{\text{track}}E_{\text{track}}+\alpha_{\text{smooth}}E_{\text{smooth}},$$

where $E_{\text{vol}}$, $E_{\text{track}}$, and $E_{\text{smooth}}$ denote the errors associated with each objective. The non-negative weights $\alpha_{\text{vol}}$, $\alpha_{\text{track}}$, and $\alpha_{\text{smooth}}$ control the relative contribution of each term and satisfy the constraint $\alpha_{\text{vol}}+\alpha_{\text{track}}+\alpha_{\text{smooth}}=1$.

#### Volumetric regularization

The myocardium is partitioned into octahedra through a bijective correspondence between epicardial and endocardial triangulated surfaces referenced to anatomical landmarks. Each octahedron is further subdivided into three tetrahedra to produce a conforming volumetric tessellation. For each octahedron, indexed by *K*, the Jacobian $J_{K}$ is the ratio of its deformed to its reference volume. Because each octahedron is the union of three tetrahedra, $J_{K}$ is the ratio of its total deformed volume to its total reference volume, equivalently the reference volume-weighted mean of the three tetrahedral dilatations. The deviatoric kinematics, meanwhile, remains resolved by the constituent tetrahedra. The volumetric regularizer penalizes departures from local volume preservation as

(2) $$E_{\text{vol}}=\sum_{K}g\left(J_{K}\right),$$

where summation runs over all octahedra and *g* is a smooth, strictly convex penalty function satisfying g(1)=0 [17]. For all computations reported herein, we use $g(J)={\left(J^{2}-J^{-2}\right)}^{2}$, which was selected after testing several alternative forms and was found to provide consistently fast convergence. Associating the Jacobian with the octahedron rather than with each constituent tetrahedron penalizes the mean dilatation over the three-tetrahedron patch, so that the volumetric term acts only on the net volume of the patch and does not directly penalize the shape change of the individual tetrahedra, in the spirit of the mean dilatation and selective reduced integration (B-bar) methods introduced to alleviate volumetric locking in low-order elements [18, 19]. Enforcement is moreover through a penalty of finite weight rather than an exact constraint, so that the formulation represents a compressible volumetric response whose stiffness is set by $\alpha_{\text{vol}}$. Since the discretization is inherited from the clinical segmentation software and standardized identically across the endocardial and epicardial surfaces, unit weighting keeps the regularization consistent from patient to patient: it acts identically on every octahedron, so its strength depends on local volume change alone and is not modulated by the differing ventricular geometry across subjects.

#### Speckle-based image registration

The tracking error $E_{\text{track}}$ anchors the corrected epicardium to physiologically plausible tissue positions by quantifying the dissimilarity between ultrasound speckle patterns at mesh vertices and the underlying image data. Sequential (frame-to-frame) and referential (end diastole to current) tracking are combined via inverse-variance weighting. Zero-mean normalized cross-correlation (ZNCC), *ρ*(·, ·), is used as the local similarity metric due to its robustness to depth-dependent gain variations inherent in echocardiographic imaging [20]. Let $P_{I}^{k}$ denote an image patch at vertex *I* and time $t^{k}$. The speckle-tracking error at time $t^{k}$ is constructed by combining both sequential and referential ZNCC as follows:

(3) $$E_{\text{track}}=\sum_{I}\left[w_{I}\left[1-\rho_{\text{seq}}\right]+\left[1-w_{I}\right]\left[1-\rho_{\text{ref}}\right]\right]A_{I}^{0},$$

where $\rho_{\text{seq}}=\rho \left(P_{I}^{k},P_{I}^{k-1}\right)$, $\rho_{\text{ref}}=\rho \left(P_{I}^{k},P_{I}^{0}\right)$, and $A_{I}^{0}$ is the reference Voronoi area associated with vertex *I*.

The adaptive fusion weight *w_I_* ∈ [0, 1] balances sequential and reference contributions based on their relative uncertainties, computed via inverse-variance weighting

(4) $$w_{I}=\frac{\sigma_{ref}^{2}}{\sigma_{seq}^{2}+\sigma_{ref}^{2}}$$

Where

(5) $$\sigma_{seq}^{2}=\frac{1-\rho_{seq}^{2}}{\rho_{seq}^{2}},\;\sigma_{ref}^{2}=\frac{1-\rho_{ref}^{2}}{\rho_{ref}^{2}}.$$

This dual-tracker approach mitigates the complementary failure modes of each method: sequential tracking accumulates drift over time as small frame-to-frame errors compound, while reference tracking degrades due to progressive speckle decorrelation [21–25]. High reference variance drives $w_{I}\to 1$, emphasizing sequential tracking, whereas high sequential variance (e.g., from poor frame-to-frame matching) drives $w_{I}\to 0$, favoring the reference template.

#### Geometric smoothness regularization

To prevent high-frequency artifacts during epicardial optimization, we incorporate a discrete Laplacian-based smoothness penalty [26, 27]

(6) $$E_{\text{smooth}}=\sum_{I}{\left\|\text{Δ}\delta {\text{v}}_{I}\right\|}^{2}A_{I}^{0},$$

where $\delta v_{I}$ denotes the displacement of epicardial vertex *I* from its original segmented position and **Δ** is the Laplacian operator on the reference epicardium. This formulation penalizes the discrete Laplacian of the vertex perturbation field, suppressing sharp features and promoting smooth, physiologically plausible epicardial geometries.

Optimization of the epicardial mesh was performed in Matlab utilizing the fminunc solver and a limited-memory BFGS (L-BFGS) quasi-Newton algorithm. The total error was minimized to ensure robust convergence across the cardiac cycle, subject to a step tolerance of 10^−6^.

### 3D Strain Analysis

Local myocardial deformation is quantified relative to the ED reference configuration for each tetrahedron using the deformation gradient **F**, which maps reference edges $G_{i}$ to their deformed counterparts $g_{i}$ via $g_{i}={FG}_{i}$ [28]. The Jacobian of deformation in Cartesian coordinates is written as *J* = det **F**. The deformation gradient admits the polar decomposition

(7) F=QU=VQ,

where **Q** is a proper orthogonal rotation and **U** and **V** are symmetric positive-definite stretch tensors [29]. The Green–Lagrange strain and the Biot strain tensors are defined as

(8) $$E^{GL}=\frac{1}{2}\left(U^{2}-I\right),\;E^{\text{Biot}}=U-I$$

where **I** is the referential identity tensor [30]. Although Green–Lagrange strain is the conventional strain measure in solid mechanics, Biot strain is frequently preferred for characterizing myocardial deformation [31]. Biot strain provides a large deformation tensorial formulation that rigorously captures 3D tissue stretch while preserving the linear, clinically intuitive scaling of traditional engineering strain. In contrast, the quadratic nature of the Green–Lagrange tensor diminishes its direct physical interpretability in this context.

Let $\lambda_{i}>0(i=1,2,3)$ denote the eigenvalues of **U**, with corresponding normalized eigenvectors $u_{i}$. These *λ_i_*’s are the principal stretches, $\lambda_{i}-1$ are the principal strains, and $u_{i}$ the principal directions in the reference configuration. The eigenvalues of the Green–Lagrange strain tensor $E^{GL}$ are $\frac{1}{2}\left(\lambda_{i}^{2}-1\right)$. Each reference principal direction **u***_i_* maps to the deformed configuration as $v_{i}=Qu_{i}$.

Principal stretches and directions are computed for each tetrahedron after optimization. During systole, the typical ordering $\lambda_{1}<\lambda_{2}<1<\lambda_{3}$ reflects tangential myocardial contraction accompanied by wall thickening. The 3D contraction pattern is visualized by tracing the direction of maximum contraction **v**_1_ through adjacent tetrahedra. This generates a dense network of discrete trajectories, with variable contraction intensity, representing the net mechanical effect of myocardial fiber shortening throughout the ventricular wall.

Because the surfaces are propagated by speckle tracking, the tracked acoustic features act analogously to fiducial markers embedded within the myocardium, so the accuracy of the computed regional strains depends more on robust feature tracking over time than on the precise anatomical definition of the initial endocardial and epicardial boundaries.

#### Global Strains for Validation

Reference myocardial strains were quantified from the CMR datasets using *Medis Suite MR* (Medis Medical Imaging, Leiden, The Netherlands). The software evaluates global strains from CMR slices (2D images) following the clinical standard [32] as 1D Lagrangian strains: Global longitudinal strain (GLS) was computed in each LAX view as $GLS=\frac{L-L_{0}}{L_{0}}$ and Global circumferential strain (GCS) was calculated in each SAX view as GCS $=\frac{C-C_{0}}{C_{0}}$ , where $L_{0}$ and $C_{0}$ represent the longitudinal arc length and short-axis circumference at the ED reference configuration, while *L* and *C* denote their respective lengths in the deformed state.

GCS was also calculated from apical long-axis (LAX) views [33]. In this case, the ventricular cross-section is assumed to be circular and the circumferential strain simplifies to the transverse diametric strain $\frac{D-D_{0}}{D_{0}}$, where $D_{0}$ and *D* are the transverse ventricular diameters at ED and the deformed state. This is evaluated at each level from base to apex, and the GCS for that image plane is obtained by averaging them.

To compute global LV strain values, LAX-derived GLS and GCS are averaged across the three longitudinal images; similarly, SAX-derived GCS is averaged across the three transversal views. The software reports the endocardial and the average myocardial strain values, where the latter is obtained internally by performing an integration across the thickness by summing the values at the endocardial, midmyocardial, and epicardial levels with weights 0.25, 0.5, and 0.25, respectively.

To perform a proper comparison with CMR, the local deformation from 3D echocardiography is also evaluated using the Biot strain tensor $E^{\text{Biot}}$ . In the reference configuration, let **a** denote the unit vector along the ventricular long axis (pointing from base to apex) and $e_{N}$ denote the outward unit normal vector to the local myocardial surface. The local longitudinal direction $e_{L}=\frac{a-\left(a\cdot e_{N}\right)e_{N}}{\left\|a-\left(a\cdot e_{N}\right)e_{N}\right\|}$ , is the normalized projection of **a** onto the local tangent plane of the surface. The local circumferential direction $e_{C}$ , is defined as $e_{C}=e_{N}\times e_{L}$ , ensuring an orthogonal local coordinate system on the surface. The local longitudinal $\left(E_{LL}\right)$ and circumferential $\left(E_{CC}\right)$ strains for each tetrahedron are calculated by projecting the Biot strain tensor along these anatomical directions using $E_{LL}=e_{L}\cdot \left(E^{\text{Biot}}e_{L}\right)$ and $E_{CC}=e_{C}\cdot \left(E^{\text{Biot}}e_{C}\right)$ . Finally, the 3D GLS and GCS for the targeted region (e.g., endocardium or full myocardium) are obtained by calculating the volume-weighted average of these local tetrahedral strains based on their undeformed reference volumes.

## Results

### Sensitivity Analysis

A sensitivity analysis was performed to examine the influence of the weighting parameters $\alpha_{\text{vol}}$ , $\alpha_{\text{track}}$ , and $\alpha_{\text{smooth}}$ on the performance of the optimization framework. The parameters were evaluated using the values {0.2, 0.4, 0.6} under the constraint that they sum to one, while maintaining a cubic patch size of *N* = 5 voxels along each spatial dimension (a 5 × 5 × 5 voxel volume), which provided a practical balance between accuracy and computational cost based on preliminary testing.

**Fig. 3:**
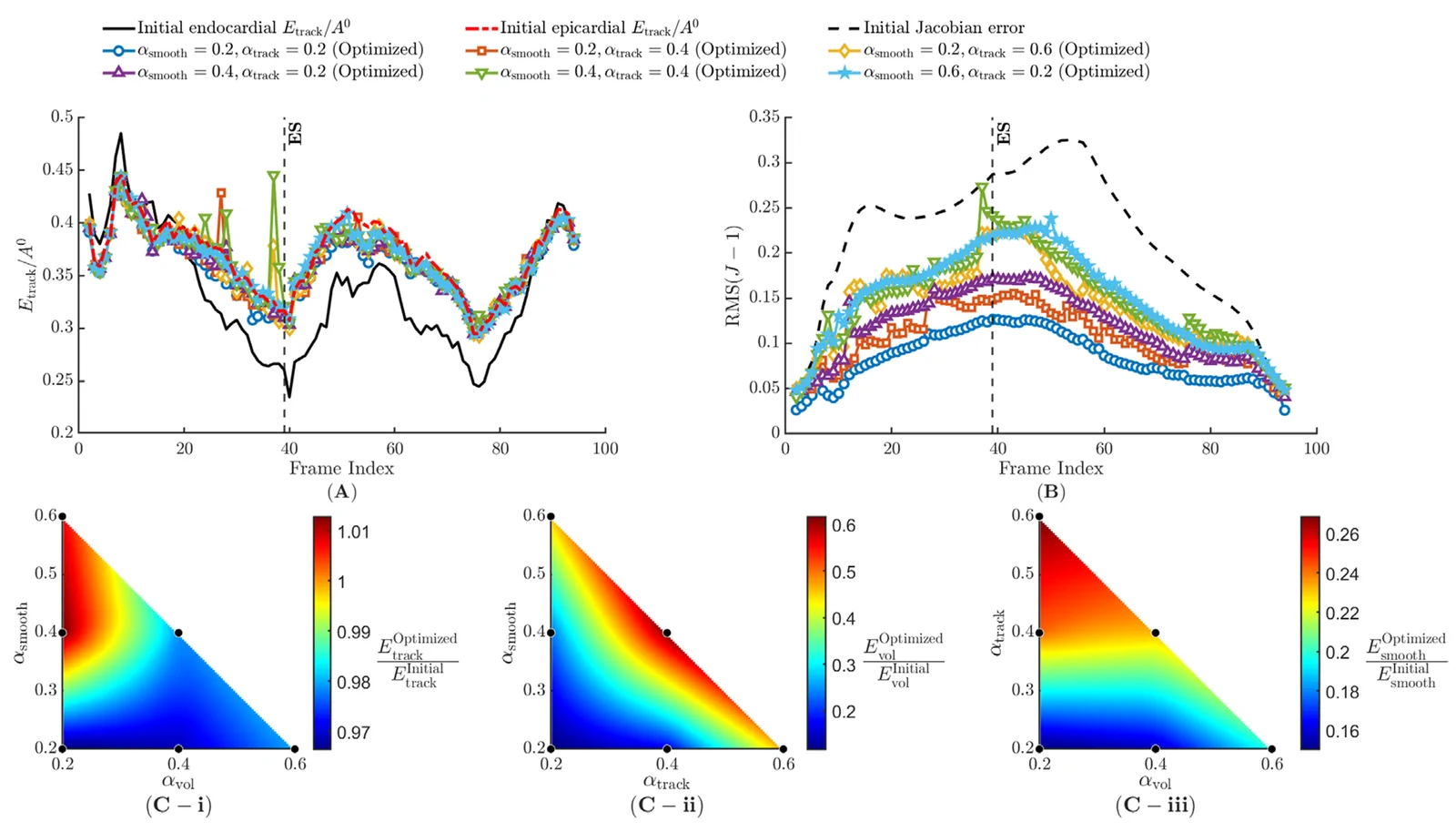
Sensitivity analysis of optimization weights $\left(\alpha_{\text{vol}},\alpha_{\text{track}},\alpha_{\text{smooth}}\right)$. **A** Area-normalized tracking error $E_{\text{track}}^{\text{avg}}$ post-optimization remains within range of the initial epicardial and endocardial segmentations across all parameter combinations. **B** Volume incompressibility, measured by rms (*J* − 1) , demonstrates that higher $\alpha_{\text{vol}}$ consistently improves volume preservation. **C** Heatmaps illustrate post-optimization improvement ratios mapped across cyclic parameter pairings: the optimized epicardial tracking error $E_{\text{track}}$ relative to the initial epicardium is shown over $\alpha_{\text{vol}}$ vs. $\alpha_{\text{smooth}}$; the optimized volume error $E_{\text{vol}}$ relative to the initial segmentation is mapped over $\alpha_{\text{smooth}}$ vs. $\alpha_{\text{track}}$ ; and the optimized smoothness error $E_{\text{smooth}}$ relative to the first optimization iteration is mapped over $\alpha_{\text{track}}$ vs. $\alpha_{\text{vol}}$

The three weights play distinct and largely complementary roles. The tracking weight $\alpha_{\text{track}}$ anchors the corrected epicardium to the speckle data. If reduced too much, it allows the volumetric and smoothness terms to pull the surface away from the image. However, as shown in Fig. 3A, the normalized tracking error $\left(E_{\text{track}}/A^{0}\right)$ remained comparable to baseline speckle-tracking values across all tested parameter combinations. The endocardium consistently exhibited lower tracking errors than the epicardium, as expected from baseline measurements. Crucially, the optimization procedure preserved original tracking fidelity without measurable loss.

The volumetric weight $\alpha_{\text{vol}}$ controls how strongly local volume preservation is imposed. Increasing $\alpha_{\text{vol}}$ progressively drove the Jacobian toward unity and reduced the departure from local volume preservation, measured as rms(*J* – 1) (Fig. 3B). Notably, this improvement was achieved while leaving the tracking error essentially unchanged.

The smoothness weight $\alpha_{\text{smooth}}$ governs surface regularity; large values over-smooth genuine wall features, while small values admit high-frequency artifacts. The heatmaps in Fig. 3C make the interplay between these terms explicit: the tracking residual is minimized at lower values of $\alpha_{\text{smooth}}$ (Fig. 3C–i), and the smoothness residual is minimized at lower values of $\alpha_{\text{track}}$ . Both metrics remained largely insensitive to increases in $\alpha_{\text{vol}}$. Because performance was stable across the entire range examined, the reconstruction is robust to the precise choice of weights and does not require delicate tuning.

This robustness directly informs the choice of operating point. We adopted $\alpha_{\text{vol}}=0.6$ and $\alpha_{\text{track}}=\alpha_{\text{smooth}}=0.2$ for all subsequent analyses, as this provides the strongest volumetric weighting available while still retaining appreciable data-fidelity and smoothness contributions. We did not pursue stronger volumetric weighting, both to avoid starving the other terms and because the volumetric term acts as a regularizer that should not be strictly driven to the incompressible limit. Even at the adopted weights, the Jacobian deviation does not vanish entirely; rather, the weighting suppresses large, non-physiological volume discrepancies introduced by independent segmentation while preserving genuine volume changes. Analogous behavior and close agreement were observed in both the healthy and PAH cases, supporting the transferability of this operating point, although the weights remain free parameters that may be tuned for datasets of differing image quality.

### Cross-Modality Validation Against CMR

#### End-Systolic Strain Comparisons

End-systolic global longitudinal strain (GLS) and global circumferential strain (GCS) derived from 3D echocardiography were compared with CMR measurements in both the healthy LV and the LV of the PAH patient (Table 1). Because endocardial strains obtained from 3D echocardiography are derived directly from the initial tracking without further modification, they provide a useful reference for assessing baseline tracking fidelity. Consistent with this expectation, endocardial strains showed strong agreement with both long-axis (LAX) and short-axis (SAX) CMR measurements. In the healthy subject, endocardial GLS and GCS from 3D echocardiography were −24.63% and −35.85% , corresponding to relative differences of approximately 2.0% and 1.4% with respect to LAX-derived CMR values, and 3.6% relative to SAX-derived GCS. A similarly close agreement was observed in the PAH patient.

In contrast, the initial pre-optimization myocardial strain estimates derived from 3D echocardiography were accurate in the healthy subject but exhibited substantial deviations in the PAH case. In this patient, myocardial strains were markedly overestimated, with relative differences ranging from 35 to 48% compared with CMR. After optimization, these discrepancies were markedly reduced. The post-optimization values from 3D echocardiography showed close agreement with the CMR measurements, with relative differences of 3.7% for GLS and below 1.0% for GCS with respect to LAX-derived values, and 3.4% relative to SAX-derived GCS. These results demonstrate a clear improvement in myocardial strain estimation following optimization, particularly in the structurally altered ventricle.

#### Dynamic Strain Trajectories

**Fig. 4:**
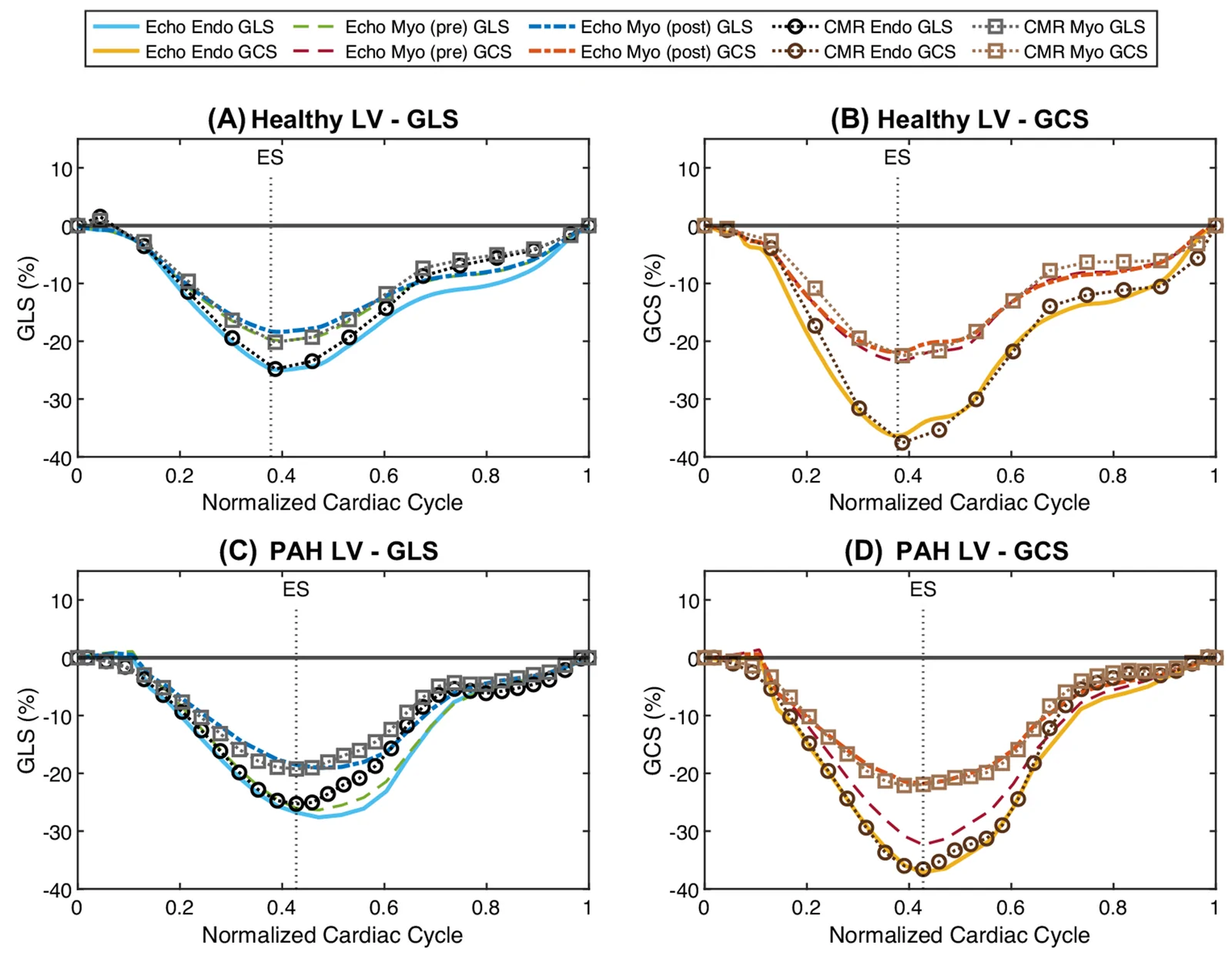
Validation over cardiac cycle. Global longitudinal strain (GLS) and global circumferential strain (GCS) over the normalized cardiac cycle for a representative healthy left ventricle (**A, B**) and a left ventricle with pulmonary arterial hypertension (PAH; **C, D**). In each panel, we compare the endocardial strain obtained from 3D echocardiography (3D Echo Endo) with the volume-weighted transmural strain computed across the endocardial, mid-wall, and epicardial layers, evaluated both from the initial epicardial segmentation (Myo Pre) and from the physics-informed epicardial reconstruction (Myo Post). These are plotted against the corresponding long-axis CMR feature-tracking estimates of endocardial and myocardial strain (CMR (LAX) Endo, CMR (LAX) Myo). Longitudinal and circumferential directions are defined relative to the ventricular long axis, and all strains are Lagrangian and referenced to end-diastole, so each curve closes over the cycle. To place the two modalities on a common timeline, we apply a two-segment piecewise-linear time warp to each trace that holds end-diastole at the cycle boundaries and maps each modality’s end-systole onto a shared normalized time, taken as the average of the echocardiographic and CMR end-systolic times (ES, vertical line); the contraction and relaxation phases are rescaled independently, and strains are reported in percent

Figure 4 displays the full-cycle strain trajectories underlying these comparisons, with the 3D echocardiography and CMR timelines aligned at end diastole and end systole via a piecewise-linear warp. For both ventricles, the endocardial GLS and GCS closely track the CMR feature-tracking curves across the entire cardiac cycle, accurately reproducing the systolic descent and diastolic recovery.

In the healthy left ventricle (Panels A and B), the pre- and post-optimization myocardial curves are nearly indistinguishable and align well with the CMR reference points. This confirms that the baseline tracking accurately captures normal physiological deformation without requiring significant correction.

The dynamic strain curves for the PAH patient (Panels C and D) highlight substantial tracking discrepancies prior to optimization. While the endocardial strains (solid lines) consistently match the CMR data (circles), the pre-optimization myocardial strains (dashed lines) markedly overestimate deformation. These curves notably overshoot the CMR reference values (squares), drifting furthest apart as the ventricle approaches end-systole. The physics-informed optimization successfully corrects these trajectories (dash-dotted lines), shifting them to closely mirror the CMR references across the entire cycle rather than only at the end-systolic peak. Because both longitudinal and circumferential components display this same behavior, the results confirm that the framework not only rectifies peak systolic errors but also restores accurate temporal dynamics across multiple strain directions in the structurally altered ventricle.

#### Algorithmic Robustness to Segmentation Variability

The robustness of the optimization algorithm to variability in epicardial segmentation was assessed by evaluating myocardial strain across three segmentation conditions: accurate (reference), over-contoured (expanded), and under-contoured (contracted) epicardial boundaries (Table 2).

**Fig. 5:**
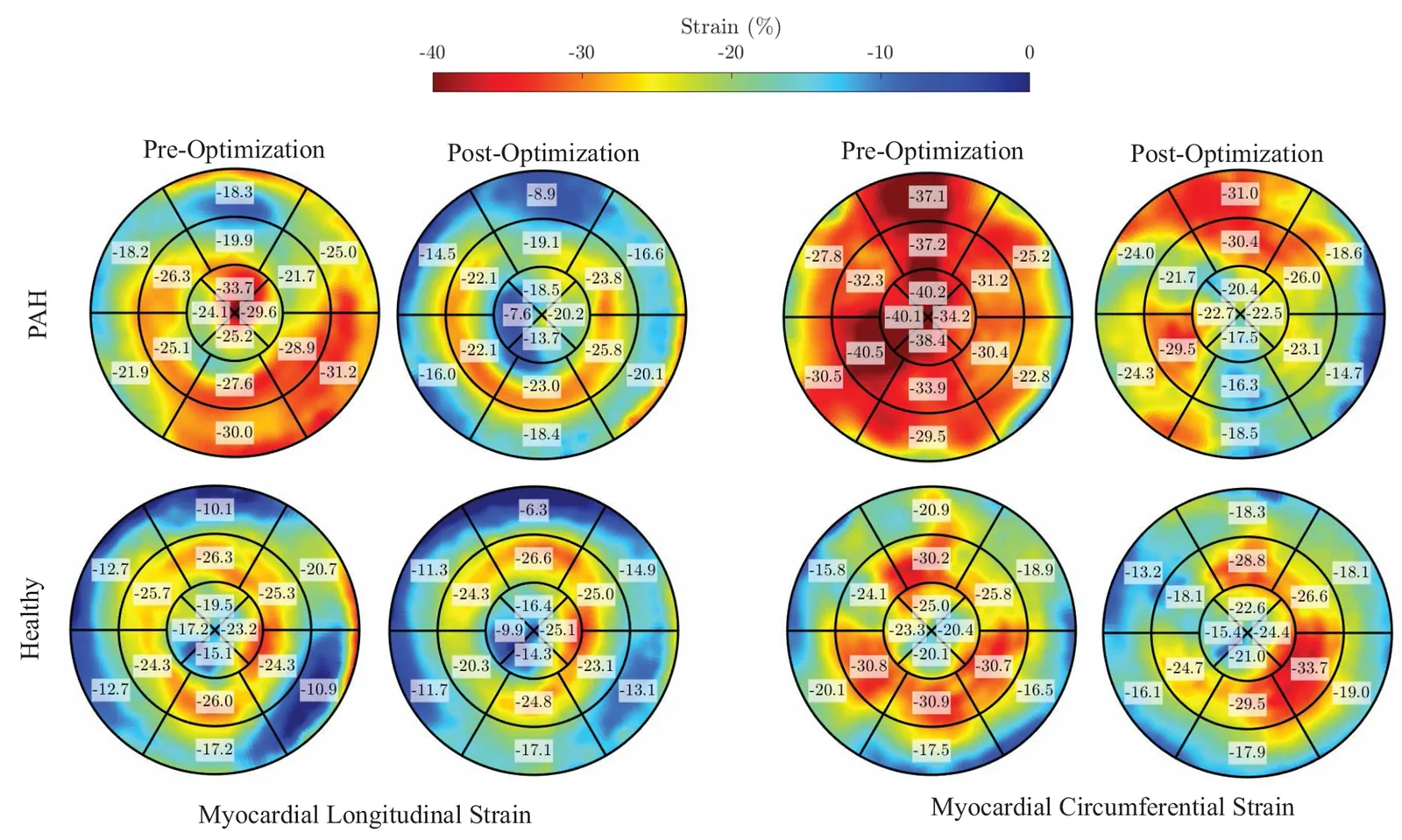
Segmental myocardial strain at end-systole (ES) before and after optimization. The bulls-eye plots compare pre-optimization and post-optimization spatial maps for myocardial longitudinal strain (left panels) and myocardial circumferential strain (right panels). Data are presented for the patient with pulmonary arterial hypertension (PAH, top row) and the healthy subject (bottom row). Pre-optimization maps represent the initial strains derived from vertex tracking prior to applying the soft volume conservation constraint

As expected, pre-optimization strain estimates were highly sensitive to segmentation inaccuracies. For instance, in the PAH patient, the under-contoured segmentation produced severely overestimated strains ( GLS = −29.43% , GCS = −37.20% ), and the over-contoured segmentation yielded similarly disparate results ( GLS = −21.59% , GCS = −23.69% ). Despite these drastic variations in the initial inputs, the optimization algorithm consistently converged toward CMR reference values across all segmentation scenarios. For example, the under-contoured GCS estimate of −37.20% was corrected to −21.63% , closely matching the corresponding LAX-derived CMR value (−21.93% ). These results indicate that post-optimization strain estimates are largely insensitive to initial epicardial contouring errors, thereby improving the robustness and reliability of myocardial strain quantification. To further illustrate this effect at a regional level, Fig. 5 compares the segmental myocardial strain distributions before and after optimization. In the healthy subject, the spatial patterns and magnitudes of both longitudinal and circumferential strains remain highly consistent, reflecting the accuracy of the initial tracking. However, for the PAH patient, the pre-optimization maps display exaggerated strain values. The post-optimization maps demonstrate how the soft volume conservation constraint dampens these high magnitudes across all segments without disrupting the fundamental spatial pattern of the myocardial contraction.

#### Advanced Principal Strain Analysis

Beyond standard global longitudinal and circumferential strains, the proposed optimization framework enables extraction of detailed, layer-specific 3D principal strain information that may provide additional clinical insight.

**Fig. 6:**
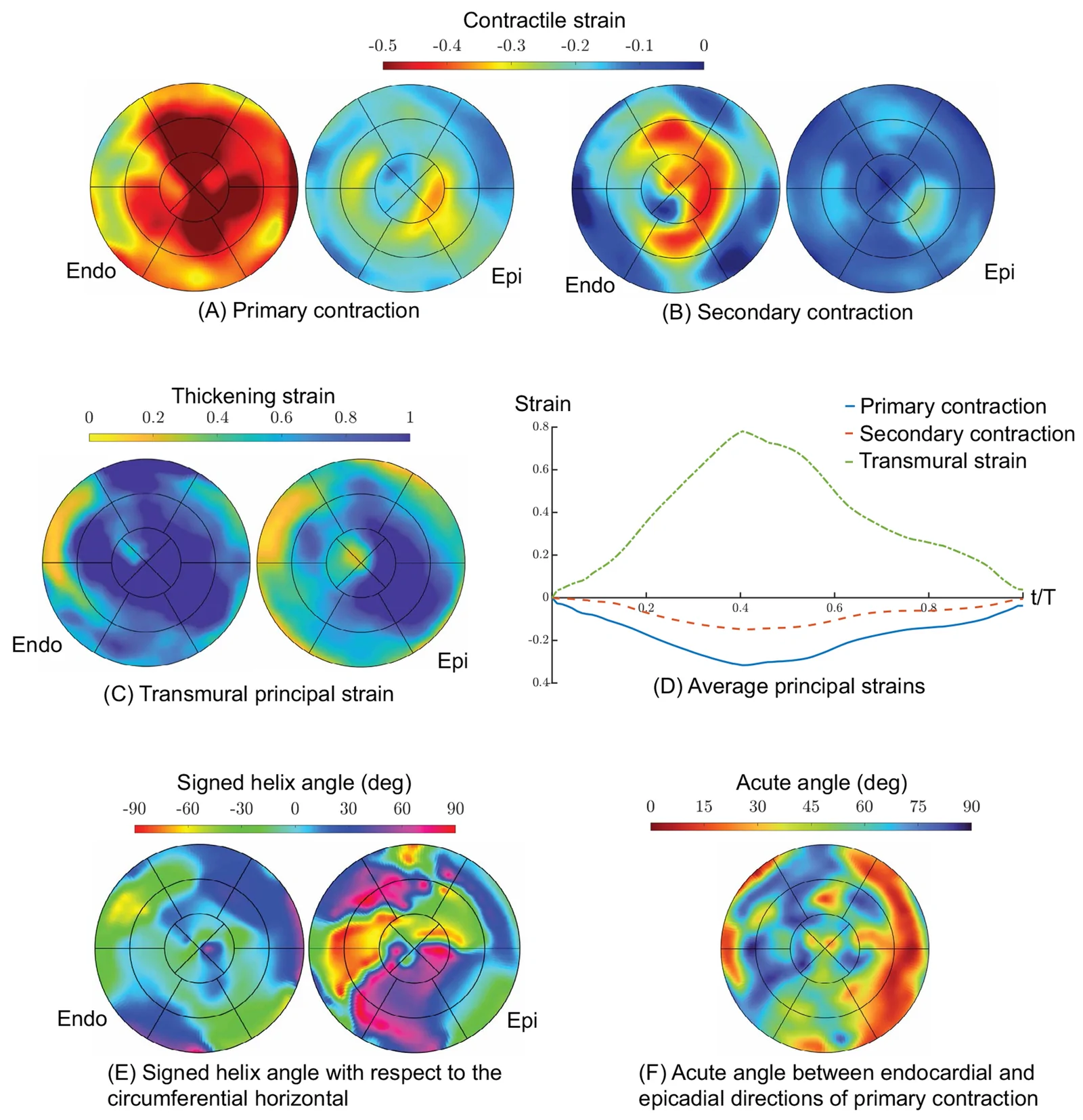
Principal strain analysis of the healthy LV. All bulls-eye maps visualize spatial distributions at end-systole using a 16-segment model over the endocardium (Endo) and epicardium (Epi). **A–C** Principal Strains: Bulls-eye maps illustrating primary contraction ( $\lambda_{1}-1$ ), secondary contraction ( $\lambda_{2}-1$ ), and transmural principal strain $\left(\lambda_{3}-1\right)$. **D** Average Principal Strains: Temporal variation of the three volume-averaged principal strains over the normalized cardiac cycle (t/T). The average is computed as a weighted mean of strains from individual tetrahedra, using their undeformed volumes as weights. **E** Primary Contraction Orientation: Bulls-eye maps showing the signed helix angle (−90° to 90°) formed by the principal eigenvector ${\text{v}}_{1}$ relative to the circumferential direction in the short-axis plane. The circumferential direction is defined via the right-hand rule (thumb pointing from apex to mitral valve); positive angles indicate ${\text{v}}_{1}$ tilts toward the base, while negative angles indicate a tilt toward the apex. **F** Transmural Alignment: A single bulls-eye map displaying the acute angle, $∠\left({\text{v}}_{\text{1}}^{\text{epi}},{\text{v}}_{\text{1}}^{\text{endo}}\right)$, between the epicardial and endocardial directions of primary contraction

This capability is illustrated by the comprehensive bulls-eye maps projected onto the AHA 16-segment model of the LV (Fig. 6), which reveal consistent physiological patterns. First, a clear transmural gradient is observed, with higher strain magnitudes at the endocardium compared with the epicardium across all principal strain components (Fig. 6A–C), as well as in their relative global contributions (Fig. 6D). Second, spatial heterogeneity is evident, with greater primary contraction in apical regions relative to basal segments. Such patterns are consistent with known physiological characteristics of LV contraction and highlight the ability of the approach to resolve spatially localized mechanical features.

In addition, the framework characterizes the spatial distribution of principal strain directions and the angular variation between endocardial and epicardial contraction directions (Fig. 6E, F), providing quantitative information on torsional and shear mechanics within the myocardial wall. These directional metrics offer complementary information to conventional scalar strain measurements by describing the 3D organization of myocardial deformation.

**Fig. 7:**
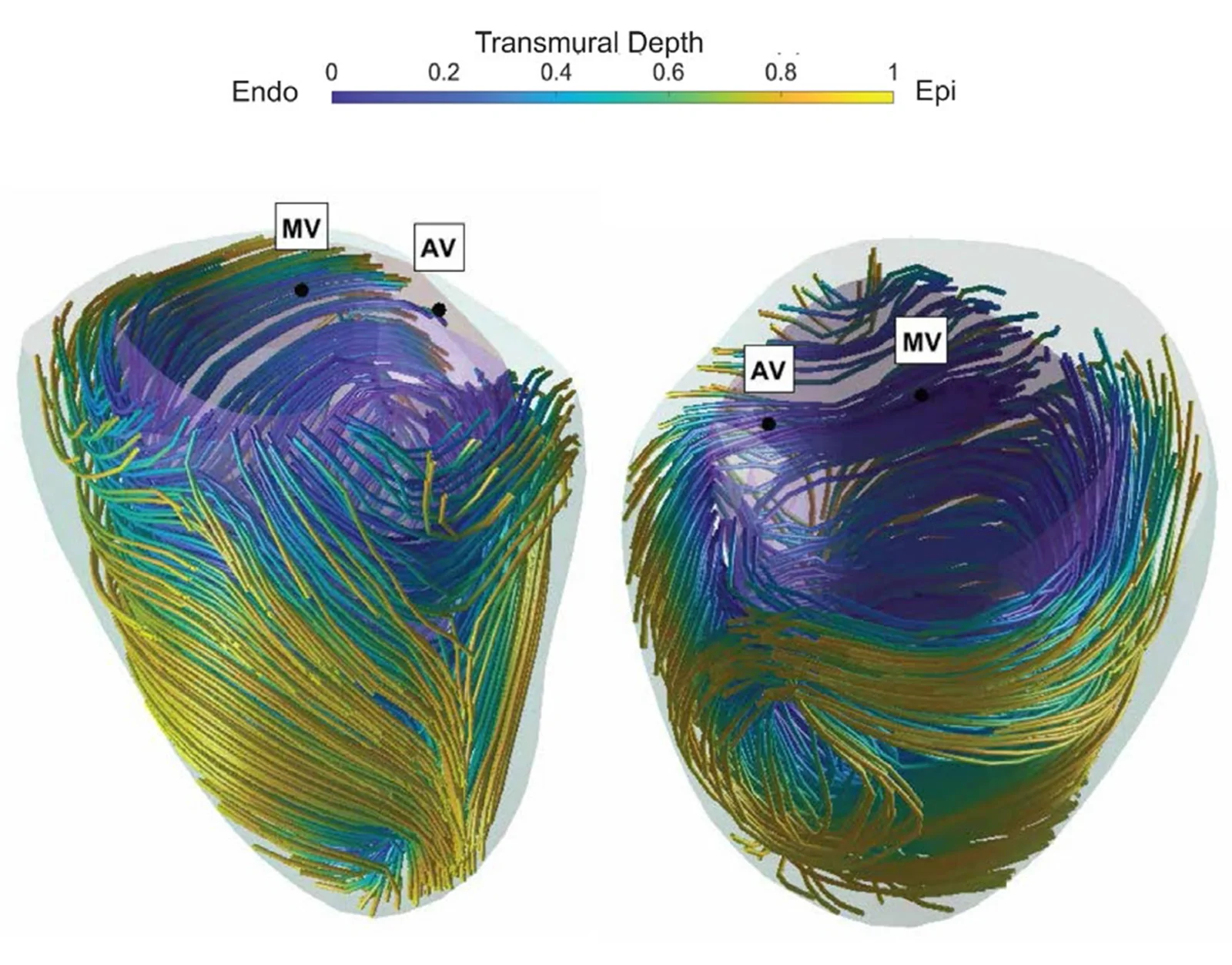
Principal strain analysis derived from 3D echocardiography. The strain lines indicate the direction of maximum contraction and are color-coded by the depth (0 on the endocardium and 1 on the epicardium). The results shown in the figure correspond to the healthy left ventricle (LV). The mitral and aortic valves are labeled as MV and AV, respectively, to aid spatial orientation

The directions of maximum contraction reconstructed by the present approach are shown in Fig. 7, displayed as continuous trajectories through the myocardial wall. Each trajectory follows the local principal direction of greatest shortening, so the field depicts how the orientation of maximal contraction varies transmurally across the wall, a characterization that is intrinsically three-dimensional and not accessible from in-plane strain on a single slice.

Finally, to provide a comprehensive spatial visualization of the myocardial deformation, a dynamic 3D transmural tractography is illustrated in Supplementary Movie S.1. This animation maps the principal direction of maximum contraction within the deformed configuration relative to end-diastole, with tracts color coded by depth from the endocardium to the epicardium.

## Discussion

Conventional clinical imaging workflows remain limited in their ability to provide a comprehensive biomechanical characterization of cardiac function. 2D echocardiography is inherently restricted to in-plane motion and is susceptible to out-of-plane speckle decorrelation, while standard cine CMR relies on multiple slice acquisitions and does not enable dense 3D material tracking without specialized imaging sequences. Furthermore, most current 3D echocardiography approaches focus primarily on endocardial motion, thereby neglecting the transmural mechanics that play a critical role in LV contraction and pumping efficiency. As a result, important aspects of myocardial deformation, such as the interaction between longitudinal, circumferential, and radial deformations across the myocardial wall, remain incompletely characterized in routine clinical imaging.

In this study, we present a physics-constrained framework based on 3D echocardiography that reconstructs deformation across the full myocardial wall, enabling direct estimation of 3D principal strains, including primary contraction, secondary contraction, and radial thickening. By integrating endocardial tracking with epicardial correction under a near-incompressibility constraint, the approach yields a physically consistent representation of myocardial deformation derived from a single 3D echocardiographic acquisition.

The sensitivity analysis further highlights the importance of balancing competing objectives within the optimization framework. A configuration with higher volumetric weighting and relatively lower tracking and smoothing weights provided the most stable and consistent results. This behavior reflects the underlying mechanics of myocardial tissue: enforcing near-incompressibility stabilizes the deformation field, while limiting reliance on image-based tracking reduces susceptibility to local imaging artifacts such as speckle noise or signal dropouts. Conversely, excessive smoothing may attenuate physiologically meaningful deformation patterns. Together, these observations underscore the value of incorporating physics-informed constraints to regularize image-based motion estimation.

### Overcoming Geometric and Transmural Limitations in Strain Imaging

Interpretation of the present results should consider the geometric limitations inherent to current strain imaging modalities. CMR techniques such as tagging, feature tracking, and displacement encoding with stimulated echoes (DENSE) are widely regarded as reference standards for assessment of myocardial deformation [1–3, 34, 35]. However, conventional slice-based acquisitions remain spatially fixed while the myocardium undergoes substantial through-plane motion during the cardiac cycle. As a consequence, myocardial tissue sampled at end-diastole does not correspond exactly to the tissue observed at end-systole, introducing systematic inconsistencies that cannot be fully corrected during post-processing [33, 36, 37]. In addition, cine CMR imaging typically relies on multi-cycle averaging, which limits the ability to capture beat-to-beat variability in myocardial mechanics [38].

2D speckle-tracking echocardiography (STE) is affected by similar geometric constraints and additional imaging limitations. Because motion tracking is restricted to a single imaging plane, out-of-plane motion leads to speckle decorrelation and inconsistent tissue sampling over time [1, 2, 39]. These effects are further compounded by the lower spatial resolution and signal variability inherent to ultrasound imaging, which may reduce tracking robustness and reproducibility.

3D STE partially mitigates these limitations by tracking motion within a volumetric dataset, enabling estimation of 3D displacement fields without relying on geometric assumptions [40–42]. However, this advantage is accompanied by reduced temporal resolution and increased computational complexity, which may limit clinical feasibility [43]. Moreover, most current 3D echocardiography approaches rely primarily on endocardial surface tracking or thin-shell representations of the myocardium [1, 44], thereby overlooking transmural strain heterogeneity.

The framework presented in this study addresses these complementary limitations by combining volumetric tracking with transmural reconstruction, enabling recovery of a dense 3D deformation field throughout the myocardial wall. This approach allows simultaneous characterization of endocardial, mid-wall, and epicardial mechanics, providing a more complete representation of myocardial deformation than methods limited to surface-based measurements.

### Myocardial Mesostructure and the Finite Strain Tensor

#### Significance of the Full Strain Tensor

A key advantage of the proposed framework is the recovery of the full six-component finite strain tensor. In current clinical practice, myocardial deformation is typically summarized using the normal strain components—longitudinal, circumferential, and radial strain. While these measures provide useful global descriptors of ventricular contraction, they do not fully capture the underlying mesostructural mechanisms that govern LV function.

At the cellular level, individual cardiomyocytes shorten by approximately 15% during systole and thicken by roughly 8%. At the organ scale, however, these microscopic deformations translate into substantially larger macroscopic changes, with directional shortening that can be up to three times greater and radial wall thickening exceeding 40% [45]. This amplification arises from shear-driven reorganization of myocardial sheetlets within the ventricular wall, a mechanism that is encoded in the off-diagonal components of the strain tensor, such as radial–circumferential shear [46]. Similarly, ventricular torsion during systole and elastic recoil during early diastole are largely governed by circumferential–longitudinal shear interactions.

By quantifying these shear components, our approach aims to capture key mechanical processes responsible for wall thickening and ventricular twisting that are not directly accessible through conventional strain metrics. Consequently, the full 3D strain tensor provides a more complete representation of LV mechanics and may offer additional insight into the structural mechanisms underlying ventricular function.

#### The Relationship Between Principal Strains and Anatomical Fibers

In cardiac strain analysis, the direction of maximum contraction—defined as the eigenvector associated with the most negative eigenvalue of the strain tensor—is sometimes interpreted as a surrogate for the local myofiber orientation. While this interpretation may hold in simplified materials composed of a single fiber layer, it does not generally apply to myocardial tissue. The myocardium is organized into superposed laminar sheets of fibers with continuously varying orientations across the ventricular wall.

Consider one such sheet and let **e***_F_* denote the unit vector along the local fiber direction and $e_{\tilde{N}}$ the unit vector along the sheet-normal direction. If $e_{F}$ were a principal direction of deformation of the right Cauchy–Green tensor $\text{C}=F^{⊤}F$ , it would satisfy ${Ce}_{F}=\lambda e_{F}$ , implying that the shear component $e_{\tilde{N}}\cdot \left({Ce}_{F}\right)$ between the fiber and sheet-normal directions vanishes. In myocardial tissue, however, this shear component is non-zero and plays an essential role in systolic wall thickening.

Experimental studies show that myocardial sheetlet planes align with directions of maximum shear and are oriented approximately ±45° relative to principal strain axes. Because the fiber direction lies within these sheetlet planes, it cannot coincide with a principal strain direction.

The principal strain directions should therefore not be interpreted as a direct anatomical surrogate for local fiber orientation. Instead, they represent the resultant directions of macroscopic contraction arising from the combined effects of fiber shortening, cross-fiber interactions, and inter-sheetlet shear. Although these directions do not coincide with the anatomical fiber architecture, they provide a functional surrogate for the dominant direction of myocardial deformation generated by that structure.

#### Linking Structure and Function

Diffusion tensor imaging (DTI) currently provides the most detailed in vivo representation of myocardial microstructure by mapping preferential water diffusion pathways within the tissue [47, 48]. Because cardiomyocytes form a branching syncytium rather than discrete cable-like fibers, DTI-derived tracts should be interpreted as representations of the dominant structural scaffolding of the myocardium rather than individual anatomical fibers [49]. Although recent technical advances have enabled in vivo myocardial DTI, practical limitations—including prolonged acquisition times and sensitivity to cardiac and respiratory motion—generally restrict these measurements to a single, quasi-static phase of the cardiac cycle [50].

In contrast, the present 3D strain analysis characterizes the temporal evolution of myocardial deformation throughout the cardiac cycle. These two approaches therefore provide complementary perspectives: DTI describes the structural organization of the myocardial tissue, whereas the proposed 3D echocardiographic framework captures the functional deformation of that structure over time. Integrating these modalities may enable future investigations to more directly link microstructural remodeling with macroscopic mechanical dysfunction.

### Limitations

This study should be interpreted as a pilot feasibility study whose aim is to establish the methodology rather than its clinical reliability, and several limitations follow accordingly. First, the method was evaluated in only two subjects as part of an initial methodological validation. Although these cases illustrate the capability of the approach, further evaluation in larger and more diverse cohorts will be necessary to establish statistical robustness and assess clinical generalizability.

Second, the accuracy of the method depends on the quality of the underlying 3D echocardiography acquisitions. Although the optimization procedure mitigates errors associated with epicardial delineation and tracking noise, inherent image quality limitations—such as acoustic shadowing, reduced signal-to-noise ratio, and signal dropout—may still influence the reliability of the reconstructed deformation field.

Finally, strain estimates were validated against CMR-derived measurements, which themselves are subject to known limitations, including through-plane motion and slice misregistration. In addition, the comparison was restricted to global strain metrics. Consequently, the reported agreement should be interpreted within the methodological constraints of the reference modality.

A direct, point-wise comparison between the 2D CMR strain and the present 3D echocardiographic strain is not appropriate, because the two modalities sample the myocardium differently: a CMR slice, once prescribed, remains fixed in the scanner frame and images whatever tissue passes through that plane as the heart moves, whereas the echocardiographic measurement is reconstructed from a geometry that tracks the myocardium itself. This single difference has both a geometric and a kinematic consequence. Geometrically, even if a CMR plane and an echocardiographic view are aligned at one reference frame, they do not remain co-located through the cycle, since one stays fixed while the other follows the moving tissue. In the long-axis views this restricts any meaningful interpretation to the in-plane longitudinal component (GLS), and even there only a spatial trend can be read rather than a quantitatively comparable value, because the two methods evaluate strain over different material domains: the CMR measurement compares the myocardial cross-sections intersecting a spatially fixed plane at end diastole and end systole, which are generally different sets of material points, whereas the echocardiographic measurement computes the deformation gradient of a fixed material region tracked between the two configurations. Under torsion this region does not remain planar, so the two methods do not characterize the deformation of the same moving tissue. The short-axis case is more strongly affected: because the ventricle translates substantially along its long axis during contraction, a fixed short-axis slice intersects different myocardium at end diastole than at end-systole, so its reference and deformed contours do not correspond to the same material tissue. A 2D short-axis circumferential strain (GCS) is therefore an unreliable reference, and a discrepancy with the echocardiographic estimate is expected, reflecting the limitation of the reference rather than an error in the present method.

A segment-level and transmural validation would further strengthen the regional claims of the framework, but this is constrained by the reference modality rather than by the method itself. The CMR data available here consist of a small number of widely spaced two-dimensional planes (three long-axis and three short-axis views), so the inter-slice sampling is coarse and the through-plane component of motion is not captured by in-plane feature tracking. 2D CMR feature tracking is correspondingly limited in regional reproducibility and does not provide an adequate quantitative ground truth for a segment-by-segment or layer-by-layer comparison [51–55]. A rigorous local and transmural validation would require ground truth in which transmural strains are known independently of any tracking algorithm, such as an in silico kinematic heart model or an in vitro phantom. A three-dimensional CMR reference with through-plane tracking, such as tagged or DENSE CMR, would mitigate the coarse inter-slice sampling and limited through-plane sensitivity of the present 2D acquisition. However, 3D speckle-tracking echocardiography is itself limited in regional and transmural accuracy and reproducibility [1], so any discrepancy against a 3D CMR reference would not be readily attributable to the reconstruction alone, since it could equally reflect variability in the echo input. Such references were not available in the present study.

### Concluding Remarks

In conclusion, this study introduces a physics-constrained framework for reconstructing myocardial deformation from 3D echocardiography across the full ventricular wall. By integrating speckle-tracking measurements with biomechanical constraints, including near-incompressibility and geometric regularization, the proposed approach enables robust estimation of transmural deformation while reducing common sources of tracking and segmentation error.

The method reconstructs the complete 3D strain tensor, allowing characterization of myocardial mechanics beyond conventional longitudinal, circumferential, and radial strain metrics. Validation against CMR measurements demonstrated strong agreement in global strain indices, while additional analyses confirmed the robustness of the framework to segmentation variability and its ability to resolve physiologically consistent transmural and regional strain patterns.

Together, these findings demonstrate that integrating image-based tracking with physics-based constraints can substantially enhance the reliability and interpretability of cardiac strain analysis. With further validation in larger populations, this framework may provide a practical pathway toward comprehensive, non-invasive assessment of myocardial mechanics from widely available 3D echocardiography, enabling richer functional characterization of the LV in both research and clinical practice.

## Supplementary Material

Video S1

The online version contains supplementary material available at https://doi.org/10.1007/s10439-026-04335-y.

## Figures and Tables

**Table 1:** End-systolic global longitudinal strain (GLS) and circumferential strain (GCS) evaluated from CMR long-axis (LAX) and short-axis (SAX) slices and from 3D Echocardiography computed from the initial segmentation (Pre) and post-optimization segmentation (Post)

Layer	Strain	Healthy LV				LV of PAH patient			
		CMR		3D Echo		CMR		3D Echo	
		LAX	SAX	Pre	Post	LAX	SAX	Pre	Post
Endocardium	GLS	−24.15		−24.63		−25.29		−26.97	
	GCS	−36.37	−34.59	−35.85		−36.59	−35.32	−37.93	
Myocardium	GLS	−19.78		−19.73	−18.30	−19.24		−26.04	−18.53
	GCS	−22.22	−23.51	−23.30	−21.85	−21.93	−22.55	−32.36	−21.78

**Table 2:** Myocardial end-systolic strains (%) from 3D echocardiography validated against CMR

LV subject	Myo- strain	CMR (LAX)	3D echo (different epicardial segmentations)					
			Good		Over-contoured		Under-contoured	
			Initial	Optimized	Initial	Optimized	Initial	Optimized
Healthy	GLS	−19.78	−19.73	−18.30	−17.96	−17.2	−22.18	−17.74
	GCS	−22.22	−23.30	−21.85	−19.67	−21.08	−28.89	−22.55
PAH patient	GLS	−19.24	−26.04	−18.53	−21.59	−18.65	−29.43	−20.27
	GCS	−21.93	−32.36	−21.78	−23.69	−20.89	−37.20	−21.63

Echo results report the initial tracking and optimized values across different epicardial segmentations

## Data Availability

The data supporting the findings of this study are available from the corresponding author upon reasonable request.

## Code Availability

The code used for the analysis in this study is available from the corresponding author upon reasonable request.
